# Agarose Gel Characterization for the Fabrication of Brain Tissue Phantoms for Infrared Multispectral Vision Systems

**DOI:** 10.3390/gels9120944

**Published:** 2023-11-30

**Authors:** Efraín Albor-Ramírez, Miguel Reyes-Alberto, Luis M. Vidal-Flores, Enoch Gutierrez-Herrera, Miguel A. Padilla-Castañeda

**Affiliations:** Applied Sciences and Technology Institute ICAT, National Autonomous University of Mexico UNAM, Ciudad Universitaria, Mexico City 04510, Mexico; albor.2020@gmail.com (E.A.-R.); luismiguelvf10@gmail.com (L.M.V.-F.); enoch.gutierrez@icat.unam.mx (E.G.-H.)

**Keywords:** phantom, agarose, tissue-like phantom, near infrared vision, multispectral imaging

## Abstract

Synthetic phantoms that recreate the characteristics of biological tissues are valuable tools for systematically studying and comprehending physiologies, pathologies, and biological processes related to tissues. The reproduction of mechanical and optical properties allows for the development and evaluation of novel systems and applications in areas such as imaging, optics, ultrasound, or dosimetry, among others. This paper proposes a methodology for manufacturing agarose-based phantoms that mimics the optical properties of healthy brain tissue within the wavelength infrared range of 800 to 820 nm. The fabrication of such phantoms enables the possibility of testing and experimentation in controlled and safe environments toward the design of new near-infrared multispectral imaging systems in neurosurgery. The results of an experimental optical characterization study indicate the validity and reliability of the proposed method for fabricating brain tissue phantoms in a cost-effective and straightforward fashion.

## 1. Introduction

The use of phantoms, or tissue-like phantoms, in the study of optical properties in the infrared spectrum is a technique that facilitates detailed research, opening the possibility of a deeper understanding of the physiologies, pathologies, and biological processes associated with tissues. In a controlled and meticulous manner, they allow us to precisely observe and analyze optical behaviors in the infrared range, showing details that could go unnoticed in less controlled conditions. This approach not only drives fundamental research in biology and medicine but also lays the foundation for the development of innovative technologies, such as the detection of anatomical structures of interest and pathological tissues through spectroscopy techniques, allowing for a noninvasive and non-ionizing diagnosis [1,2]. Examples include optical coherence tomography (OCT) employed for the identification of various structures, such as certain types of glioblastomas, laminar structures present in the cerebral cortex, or subcortical nuclei [3,4]. The use of tissue similes in this technique could help obtain high-resolution images of biological tissues, allowing for the validation and adjustment of systems before clinical application. Laser Doppler flowmetry (LDF) capable of measuring microcirculatory flow using the Doppler effect [5] could benefit from the use of tissue similes to verify the accuracy and sensitivity of the equipment by simulating specific tissue conditions, ensuring clinical reliability. Additionally, they provide a controlled environment for training healthcare professionals in device placement and results interpretation before application on real patients. Diffuse optical tomography (DOT) implemented in the generation of retinotopic mapping and the analysis of muscle physiological functions [6] benefits from the use of tissue similes for the calibration and validation by simulating specific tissue conditions, ensuring measurement accuracy in clinical settings. They are valuable in training healthcare professionals, allowing for practicing and refining the techniques before applying them to real patients, contributing significantly to optimizing the design and configuration of DOT devices, improving the accuracy and relevance of the obtained images. Laser speckle contrast imaging (LSCI) employed to identify and quantify the blood flow by analyzing the speckle pattern [7] could benefit from tissue similes to calibrate and validate equipment by simulating specific tissue conditions, ensuring measurement accuracy in clinical settings. In research studies, tissue similes in LSCI are fundamental for conducting controlled and repeatable investigations, providing a means to understanding the interactions between structural light and different tissues under specific conditions. Although their utility may vary depending on the specific LSCI approach, in general, these models contribute to refining the technique and improving the interpretation of blood flow images. Additionally, there are other methodologies that involve superficial visualization, interstitial measurement through an optical fiber, and even photodynamic therapy [8]. These spectroscopy techniques operate in the near-infrared region (NIRS), used for diagnosis and clinical monitoring. These methods take advantage of the optical absorption and scattering properties of hemoglobin and deoxyhemoglobin, allowing the detection and visualization of structures of interest, such as the presence of tumors or vascularized tissues, and providing hemodynamics information, such as oxygenation and blood flow. In this last aspect, optical markers based on hemodynamics monitoring suffer from the relevant limitation that the optical properties of vascular transporting hemoglobin and its surrounding tissue may affect the noise-to-signal ratio in the measurements, making the detection of blood flow difficult. Therefore, extensive experimentation and system evaluation are necessary before being implemented in patients, seeking to reduce measurement errors and avoid tissue damage.

To our knowledge, the optical characterization of brain tissue phantoms in the infrared spectrum has been rarely addressed. Previous works are limited to reporting chemical, mechanical, or acoustic properties. Developing methodologies that allow us to emulate optical properties in brain tissue within the infrared range is important. This is useful, especially for systems that are aimed at tissues that are difficult to access, such as tumors or brain tissue anatomies and pathologies. Consequently, it allows for carrying out more and better studies in a controlled environment, implying exhaustive testing. Moreover, it can even be used for calibration and training [9,10,11].

Different methodologies reported in the literature have been used to make tissue similes. In general, such methodologies can be classified into two types according to the material used for the manufacture: (1) those made with polymers and (2) those made with biological materials. Examples of the former ones are polydimethylsiloxane (PDMS) [12], silicone, polyacrylamide gel (PAA) [13], polyvinyl chloride gels (PVC), styrene-ethylene-butylene-styrene (SEBS) [14,15], and polyvinyl alcohol gel (PVA); in the case of biological materials, gelatin and agarose are commonly used in many applications [16,17].

In general, using biological materials, such as collagen, gelatin, or agarose, has demonstrated chemical and structural similarities with real tissues. Furthermore, such materials provide some benefits, such as flexibility, deformability, and similar responses to biomechanical stimuli. On the other hand, the environment created by biological materials is favorable for cell interaction and proliferation, facilitating cell appearance and differentiation. Therefore, biological materials promote a more biocompatible response than many synthetic polymers [17].

Within biological materials, the use of biological hydrogels for the fabrication of tissue mimics is aimed at applications in areas such as imaging, optics, ultrasound, or dosimetry, among others. In addition, biological hydrogels are suitable for the characterization of multispectral imaging systems. Its use is due to its high-water content, resembling cell tissue, in addition to its compatibility with other organic elements, such as fluorophores or optical markers, extending its usefulness in many medical areas [8]. Within these hydrogels, agarose is a widely used material for the manufacture of brain phantoms, also given its mechanical properties, which can be adjusted by manipulating the concentration of agarose with respect to the volume of water, reporting concentrations in the range of 0.6% to 1%, to resemble the mechanics of brain tissue [17,18,19,20].

The present work proposes a methodology for manufacturing phantoms that mimic the cerebral cortex tissue’s optical properties using agarose with milk mixtures as a simile.

Previous studies have reported the use of powdered milk as a favorable component for mimicking the properties of soft tissues [21,22,23]. Among the reported characteristics are its scattering and absorption properties; when suspended in a medium, it can act as a scattering agent. Additionally, its composition, including vitamins, minerals, and transgenic fats, exhibits absorption properties in the infrared spectrum. Other studies indicate that powdered milk can be blended with other elements to tailor the properties of phantoms as needed. This provides flexibility in creating a wide range of tissue simulants. A significant advantage of using powdered milk lies in its availability and cost-effectiveness. This renders it a practical choice, particularly in research environments in which substantial quantities of phantoms are required for experimentation.

The availability of such phantoms will pave the way for designing and characterizing novel infrared multispectral imaging systems for cerebral hemodynamics monitoring, such as directly monitoring the intracranial site of the patient [24,25]. As far as we know, this is the first work that reports the optical characterization of a brain tissue-like phantom within the infrared range (i.e., 800–820 nm) using agarose.

## 2. Results and Discussion

In the methodology, a protocol is proposed for elaborating phantoms with optical properties resembling brain tissue. The optical parameters required for determining if the manufactured phantom mimics the tissue’s optical properties are the reduced scattering coefficient, $\mu_{s}^{\prime }(\lambda )$, and the absorption coefficient, $\mu_{a}(\lambda )$, both being the most used for tissue characterization, which is explained in the next section.

Vegetable milk was used as a scattering agent because it is inexpensive, simple to prepare, and has good optical properties that have been reported for the manufacture of phantoms [26]. In contrast with others scattering agents, such as white nanoparticles, like titanium dioxide or aluminum oxide, as well as polymer or lipid microspheres, they require a calibration process during production, with Intralipid being the most reported [27].

Different materials, such as inks or coffee, are reported as absorbing agents due to their melanoidin content [5,14,28,29]; moreover, it was observed that milk contains absorbing agents, such as lactose and trans fats, among others [30,31,32]. This indicates that milk is a good candidate agent for being used to recreate the properties of the tissue in the NIR range.

### 2.1. Characterization Results

**Figure 1:**
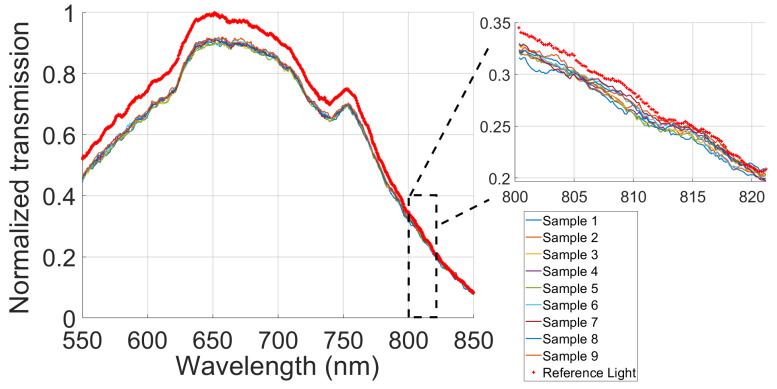
Comparative analysis of the normalized transmission light of nine agarose samples and the reference light.

Before carrying out the gel characterization with milk, an analysis was performed on the light interaction with nine agarose samples within the 800–820 nm range in our experimental system. The objective of this analysis was to verify the agarose’s quality as a matrix that would contain the scattering and absorbing agents. This implies identifying low values of the optical properties of the material. Figure 1 plots the resulting normalized transmission of light in each sample, represented by lines of varying colors. For visualization, the reference light is indicated by a red, dotted line. The presented figure depicts the interaction of light with agarose across a broad range of wavelengths, with a particular emphasis on the infrared range of interest spanning from 800 to 820 nm. In the near-infrared range, the transmission of light can be seen passing through the agarose slices closely aligned with the reference light, which implies a high transmittance.

**Figure 2:**
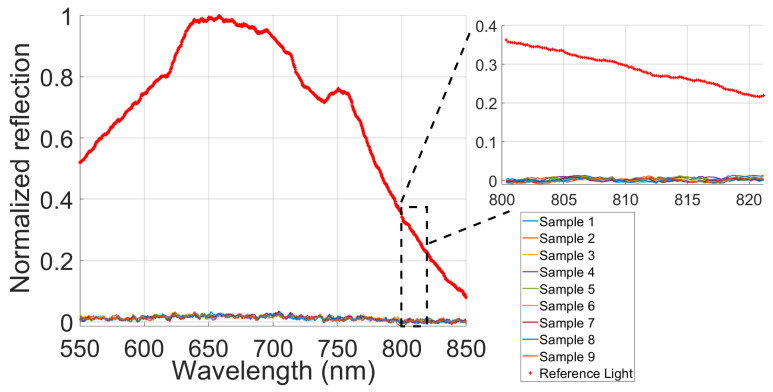
Comparative analysis of the normalized reflection light of nine agarose samples and the reference light.

Similarly, Figure 2 shows a normalized reflection close to zero in each sample, indicating a high transmittance and low reflectance in the agarose matrix. This indicates that the contribution to the absorption and scattering coefficients will be minimal and only depend on the added elements. These results, combined with the applications shown in previous works [31,32,33], indicate that agarose is suitable for recreating soft tissue-like properties such as the brain [31,32,33]. Furthermore, the consistency of light’s behavior across all nine samples highlights the repeatability of the gel fabrication process using the established protocol.

The present study employed an agarose concentration of 0.7% for the nine samples analyzed. The literature has reported a range of concentrations that mimics the mechanical properties of the brain, with an average value of 0.6% for agarose concentration [17,18,19]. However, considering our experimental configuration, a concentration of 0.7% was selected to ensure an enhanced sample stability during the measurement. This decision was based on the empirical observation that the 0.6% sample could detach from the cartridge and potentially damage the optical instruments. Conversely, the 0.7% sample exhibited good adherence to the cartridge and was, therefore, selected for the analysis.

**Figure 3:**
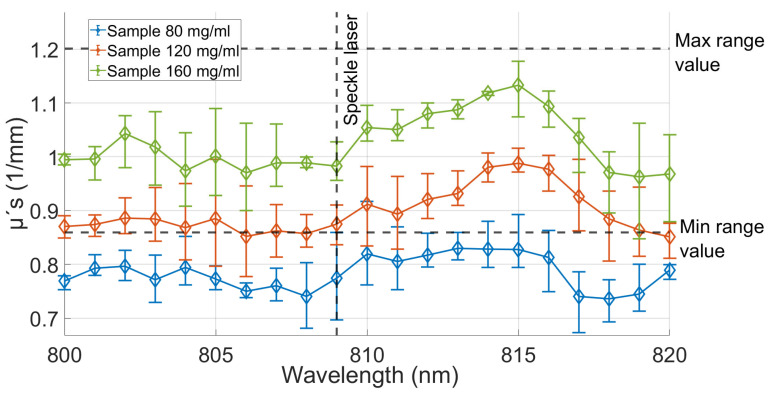
Reduced scattering coefficients of *n* = 3 samples for each concentration. Samples of the 0.7% agarose with concentrations of 80, 120, and 160 mg/mL of milk were analyzed within the range of 800 to 820 nm.

**Figure 4:**
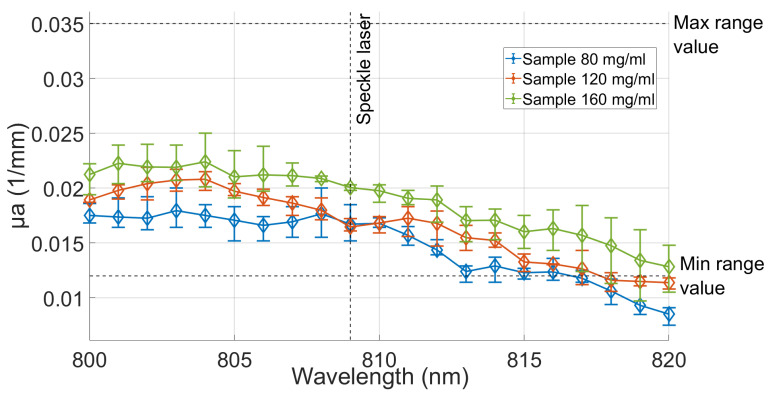
Absorption coefficients of *n* = 3 samples for each concentration. Samples of the 0.7% agarose with concentrations of 80, 120, and 160 mg/mL milk were analyzed within the range of 800 to 820 nm.

In order to characterize the agarose with milk, various samples were prepared using different milk concentrations until the desired values of $\mu_{s}^{\prime }$ and $\mu_{a}$ were achieved, as illustrated in Figure 3 and Figure 4. The figures show the calculated values for each of the coefficients for the three different concentrations of milk, namely, 80 mg/mL (green line), 120 mg/mL (orange line), and 160 mg/mL (blue line), while maintaining a constant 0.7% agarose concentration. The results obtained from these samples were compared to the established ranges for the coefficients $\mu_{s}^{\prime }$ (0.859–1.201 (1/mm)) and $\mu_{a}$ (0.012–0.035 (1/mm)) of real tissue [34,35], which are represented by the two horizontal dotted lines indicating the maximum and minimum values of the coefficients. Three samples were produced for each concentration, and the transmittance and absorbance were measured within the 800–820 nm wavelength range. These values were then utilized to calculate the coefficients using the IAD program [36], previously employed in similar studies [28,29,34,35,36,37,38,39,40,41]. Each sample was tested on the same day it was prepared; once the measurements were completed, the samples were disposed.

Figure 3 and Figure 4 present the primary outcomes of our study. These figures depict the mean values of the three samples for each concentration, as well as their corresponding minimum and maximum values. The measurements were taken at wavelengths that were spaced 1 nm, with particular attention given to the data obtained at 809 nm, which is intended as the expected excitation wavelength for the design of multispectral vision systems tailored for brain tissue monitoring applications, such as vascular flux monitoring through laser speckle contrast imaging (LSCI) [7].

In the instance of the absorption coefficient $\mu_{a}$, it was determined that the acquired milk concentration possesses satisfactory optical absorption properties within the 800–812 nm range, with an emphasis on 809 nm, to attain the $\mu_{a}$ values of the brain’s gray matter, as depicted in Figure 4. This finding negates the necessity for the utilization of absorbent agents.

**Figure 5:**
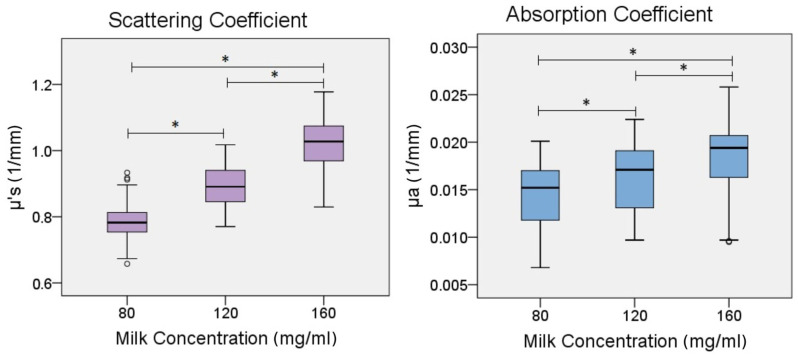
Observed scattering and absorption optical coefficients $\mu_{s}^{\prime }$, and $\mu_{a}$ of samples at different milk concentrations. * Indicates significant statistical differences of the observed optical coefficients between pairs of observations of milk concentrations.

Next, with the aim of confirming the effect of the different milk concentrations on the resulting scattering and absorption coefficients, a series of two analyses of variance (ANOVA) were carried out over the resulting samples of $\mu_{s}^{\prime }$ and $\mu_{a}$, following the experimental design of three concentrations (80, 120, and 160 mg/mL). The ANOVA confirmed a main factor effect of milk concentration for both coefficients, with F (2, 1104) = 128.313, *p* < 0.0001 for $\mu_{s}^{\prime }$ and with F (2, 1104) = 1450.834, *p* < 0.0001 for $\mu_{a}$. Then, two subsequent post hoc Bonferroni tests confirmed significant differences over the optical coefficients computed over the phantom samples at different wavelengths in the range of (800–812) nm, among every milk concentration, with significantly higher mean values for 160 than 120 mg/mL and 120 than 80 mg/mL, as shown in the boxplots of Figure 5, thus indicating the influential role of the milk concentration in the optical characterization of the brain tissue agarose phantoms. The statistics tests were performed in SPSS v.20.

A summary of the descriptive statistics results is presented in Table 1. It shows the average values of the reduced scattering and absorption coefficients $\mu_{s}^{\prime }$ and $\mu_{a}$ for real tissue. These values correspond to the 809 nm wavelength and the milk concentrations of 80, 120, and 160 mg/mL.

On the basis of the obtained results, it was determined that a concentration of 160 mg/mL is appropriate due to all coefficient values falling within the predetermined ranges. In contrast, the other concentrations exhibited resulting values outside of the reference range, particularly in the case of $\mu_{s}^{\prime }$.

Finally, to ensure that the concentration of 160 mg/mL was adequate, the repeatability of the values of the two optical coefficients was evaluated by manufacturing 12 samples with a concentration of 160 mg/mL and 0.7% agarose, and the coefficient of intraclass correlation (ICC) for each data set belonging to $\mu_{s}^{\prime }$ and $\mu_{a}$, where the ICC value of 0.9883 and 0.9892, respectively, is presented. Therefore, this indicates a high consistency between each sample for both coefficients and, consequently, demonstrates the reliability of the proposed method for fabricating brain tissue-like phantoms for imaging and optical applications in the near-infrared range.

**Figure 6:**
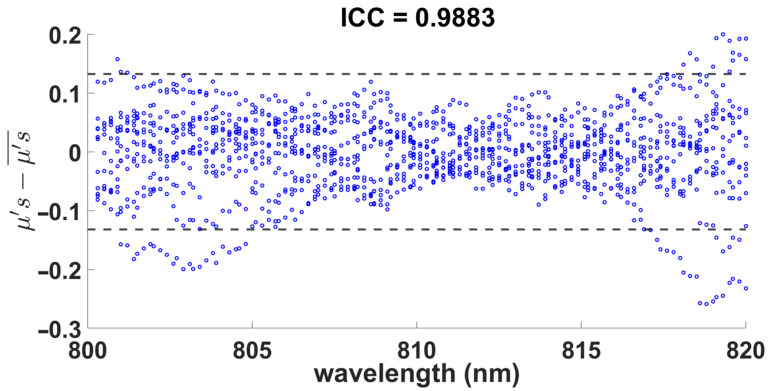
Reduced scattering coefficients of the 0.7% agarose with 160 mg/mL milk within the range of 800 to 820 nm.

**Figure 7:**
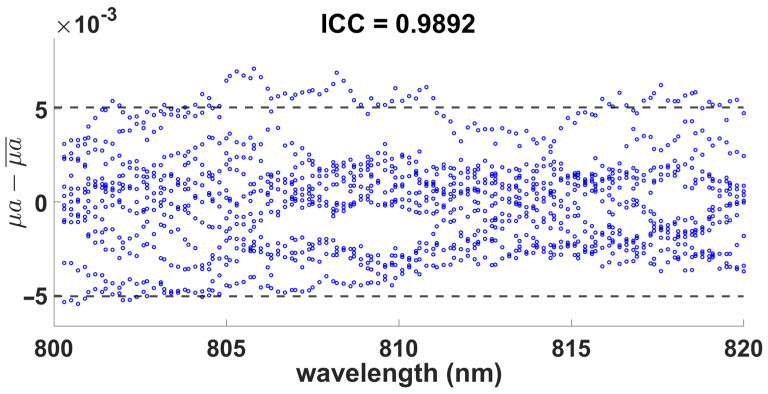
Absorption coefficient of the 0.7% agarose with 160 mg/mL milk within the range of 800 to 820 nm.

Figure 6 and Figure 7 show the plots of the limits of agreement with the mean (LOAM), where each point indicates the difference in the data with respect to the mean of the samples at each wavelength. As shown, the dispersion of the differences of the optical coefficients in both figures and its corresponding mean value presents a good agreement between the measured samples, with very few outliers outside the limits indicated by the horizontal dotted lines, which corresponds to one standard deviation representing 95% of the LOAM. Hence, the dispersion of the differences within limits complements the observed ICC scores and confirms the high repeatability of the sample measurements and their corresponding optical parameters estimation, thus demonstrating the validity and reliability of the proposed method.

### 2.2. Discussions

The results of absorption and scattering coefficients in genuine tissue samples are documented in the literature, and their values vary depending on the kind of tissue and the wavelength they interact with. The coefficients reported for a specific tissue on the same wavelength may exhibit variations across authors, and optical properties tend to be reported at specific wavelengths rather than over a broad spectrum [38,39,40].

Therefore, a range of the coefficients $\mu_{s}^{\prime }(\lambda )$ and $\mu_{a}(\lambda )$ of the gray matter of the brain tissue was defined at the wavelength of 800 nm, from 0.859 to 1.201 (1/mm) for $\mu_{s}^{\prime }$ and from 0.012 to 0.035 (1/mm) for $\mu_{a}$, as it is the closest to the desired wavelength (809 nm) for the LSCI system, taking as a reference the data reported in [34,35], which present tissue coefficients in a greater spectrum. The data were verified by comparing the coefficients at the wavelengths reported in [38,39,40], finding consistency in the ranges defined in [34,35].

Expanding such results, in our case, we introduced in this paper an optical characterization of a tissue-like phantom to replicate the optical characteristics of gray matter, which is the cortical surface tissue of the brain, within the 800–820 nm range. The primary objective of this method is to facilitate experimental investigations into the characterization, evaluation, and calibration of spectroscopy systems that operate in the near-infrared spectrum, with a specific focus on their application in the clinical field of neurology.

The parameters obtained from each sample may exhibit variations due to using a milk product intended for the food industry. This implies a low level of quality control in the size of the manufactured particles compared to other agents subjected to rigorous control during particle production. However, our results indicate that this does not pose a problem, as the parameters fall within the range of coefficients for real tissue for all samples with a 160 mg/mL concentration. Moreover, the behavior of the coefficients across the spectrum is consistent, as evidenced by the dispersion of the differences of the estimated optical coefficients against the mean values at specific wavelengths. The results of the intraclass correlation coefficients indicate a high level of repeatability in the manufacture and measurement of the samples, with a low difference between the coefficient results. Such correlation coefficients exhibit a very high value, close to 1, implying high consistency in the measurements and, thus, the fabrication of the tissue samples.

Despite the observed discrepancies among the individual samples, the phantom effectively fulfills the purpose of serving as a substitute for brain tissue in conducting experiments within a regulated setting within the scope of optical characterization of infrared imaging. This is particularly noteworthy given that the differences in the coefficients of brain tissue reported by various authors in the literature exceed the variations observed in the phantom [34,35,38,39,40].

The most common results are presented at a few specific wavelengths, such as 850 nm and 670 nm, rather than performing optical tissue characterization over a broad spectrum [38,39,40]. This practice poses a challenge in material characterization when a comparison at an unreported wavelength is sought. We used the optical properties reported at the wavelength of 800 nm [34,35], the closest being 809 nm, which is intended to be the expected excitation wavelength for designing multispectral vision systems.

Because of the low absorbance of gray matter, the absorption characteristics of milk, combined with the low absorbance of agarose, are adequate for simulating tissue. While there is a noticeable rise in the absorption coefficient, as depicted in Figure 5, with an increase in milk concentration, it is insufficient to attain higher absorption values and replicate other tissue types. Therefore, including an absorbing agent, such as coffee, would be necessary. However, milk is an effective scattering agent within the designated near-infrared range.

To conduct the optical characterization of agarose gels, each sample was promptly analyzed upon preparation and, subsequently, discarded upon completion of the test. No analyses were conducted on the samples after the passage of hours or days. As a result of its biological composition, the principal disadvantage of this phantom is its exceedingly restricted lifespan, which does not exceed one week when stored in refrigeration. This is due to the gel’s dehydration and the proliferation of bacteria and fungi. However, these limitations do not impede the utilization of the phantom for experimental purposes. Furthermore, they present an opportunity to expand this study toward the characterization of the gels, incorporating elements that can regulate or diminish bacterial proliferation and enable the reuse of dehydrated gels.

Also, a limitation of these results is not knowing yet the depth achieved by this specific wavelength, which will be helpful to elucidate how it will interact with the brain tissue. This will define how it will interact with blood vessels and features within the brain tissue.

## 3. Conclusions

In this paper, we introduced an optical characterization of a tissue-like phantom that is both cost-effective and straightforward to prepare to replicate the optical characteristics of brain tissue, within the wavelength infrared range of 800 to 820 nm, by means of agarose with milk mixtures as a tissue simile. Our results indicate that it is feasible to fabricate gray matter tissue-like phantoms with optical properties akin to cortical brain tissue at such a near-infrared range using milk concentrations of 160 mg/mL in a reliable and repeatable fashion. Such results will pave the way for experimental research in simulated environments toward the design and characterization of novel infrared multispectral imaging systems for clinical applications in neurology, such as for cerebral hemodynamics monitoring, directly monitoring the intracranial site of the patient, among others.

## 4. Materials and Methods

### 4.1. Preparation of Agarose Tissue Simile

Agarose powder (Agarose Basic, IBI Scientific, Dobuque, IA, USA) and powdered vegetable milk (Nutri Rindes, Nestle) were used to mimic the tissue. The manufacturing protocol was as follows.

#### 4.1.1. Preparation of Vegetable Milk Solution

1. Heat a quantity L (mL) of distilled water until it reaches a temperature of approximately 70 °C.
2. Add a quantity m (g) of instant milk powder to the hot water. Stir using a magnetic stirrer until complete homogenization is achieved.

#### 4.1.2. Tissue Simile Preparation

1. In a glass container, the quantities w (g) of agarose and v (mL) of distilled water should be combined. The amount of agarose will depend on the desired concentration percentage, $c_{a}$. The value is determined utilizing Equation (1).
  (1) $$c_{a}=\frac{w}{V_{T}}\times 100,$$
  with $V_{T}=v+V_{L}$, where $V_{T}$ (mL) is the total volume of the phantom, and $V_{L}$ is the volume of milk.
2. On a magnetic stirrer, heat the water with agarose up to the melting temperature, approximately 75 °C, while stirring.
3. With the use of a syringe, add a volume $V_{L}$ (mL) of the prepared milk into the heated and stirred distilled water with agarose, after the desired temperature has been reached. For a minute, continue heating and stirring.

The quantity of milk required will depend on the size of the phantom (total volume of the preparation $V_{T}$ (mL)) to be manufactured.

The amount of milk is determined as follows in Equation (2).

(2) $$M=\frac{C_{L}V_{L}V_{T}}{L},$$

where $C_{L}\left[\frac{g}{mL}\right]$ is the concentration of milk contained in the phantom.

#### 4.1.3. Agarose Phantom Sample Preparation

**Figure 8:**
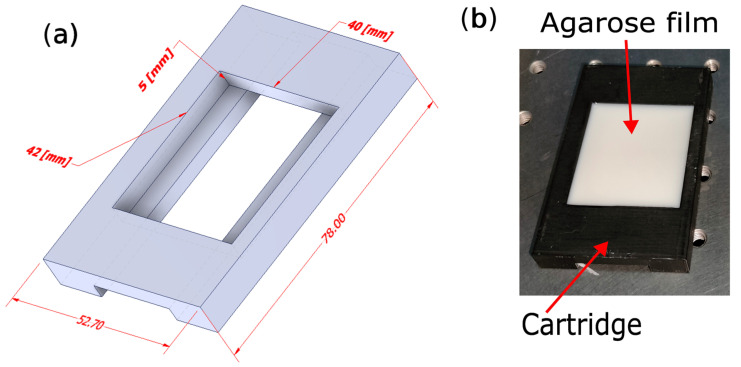
(**a**) The printing model of the cartridge is specifically designed to facilitate the pouring of the sample, (**b**) followed by the subsequent transition to the characterization stage utilizing the agarose lipid emulsion sample.

1. Once the preparation has been heated and agitated, remove the sample and allow for it to cool to a temperature of approximately 60 °C.
2. Pour the sample into a container and allow for it to cool to room temperature (see Figure 8b).

A set of 52 × 78 mm containers (42 × 40 × 5 mm of tissue sample volume) was fabricated for this project, in 1.75 mm diameter PLA (polylactic acid) filament material with a density of 1.24 $g/{cm}^{3}$, and heat the material to 210 °C, using additive manufacturing with a commercial 3D printer (Original Prusa™ i3 MK3S 3D printer, Praga, Czech Republic) with a nozzle diameter of 0.4 mm. The printer was configured to utilize a rectilinear pattern fill with a 20% ratio and a print speed of 200 mm/s, controlled using PRUSA Slicer software 2.7.0.

In Figure 8a, the lower orifice accommodates a 25.4 × 76.2 mm slide (1.0–1.2 mm thick) that is used for the purpose of pouring the sample and, subsequently, extracting the slide after the gel has solidified.

### 4.2. Optical Characterization of Agarose Tissue Simile

#### 4.2.1. Optical Parameters Estimation

With the aim of determining the adequate concentration of milk, an optical characterization was carried out over a set of tissue phantom samples by means of an arrangement that allows for capturing the reflected and transmitted light of the sample films. Then, because of its reported results, the method of inverse adding–doubling (IAD) was used to estimate the absorption and reduced scattering coefficients [6].

The IAD method calculates the transmittance and absorbance by solving the radiative transport equation using the adding–doubling method. The results are compared with the measurements made for transmittance and absorbance. From the comparison, the parameters are adjusted to approximate the theoretical values with those obtained in the measurements [36,37].

The IAD method is based on using an integrated sphere for the transmittance and reflectance measurements, as well as considering a sample with a uniform layer, with a small thickness and an area extensive enough in comparison with the entrance port of the sphere, to be regarded as an infinite surface [36].

With the purpose of having reliable repeatability in the measurements, an industrial assembly was developed, which provided us with greater control of external noise and facilitated the handling of the samples, ensuring compliance with the conditions required for the IAD method.

#### 4.2.2. Experimental Set-Up

**Figure 9:**
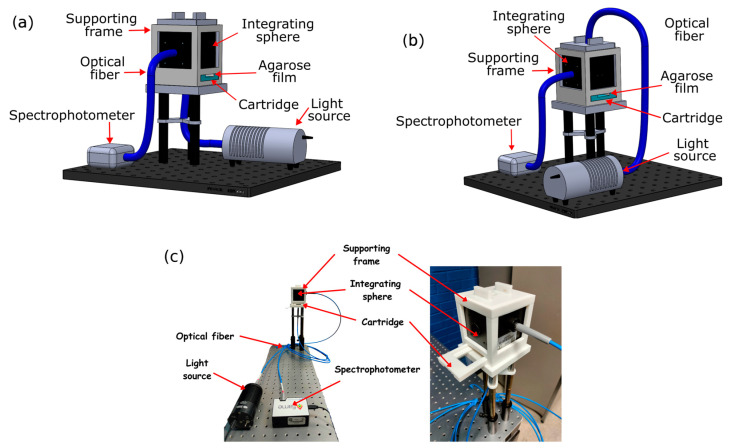
Optical arrangement for measurement of the (**a**) transmittance and (**b**) reflectance measurements; (**c**) physical experimental set-up for optical characterization.

In Figure 9, diagrams, from the side view, of the optical arrangement used to measure light in the reflection and transmission modes are shown. The sample is mounted with its horizontal surface in one of the printed cartridges (see Figure 1). The PLA mount was designed with two compartments and a port where an optical fiber is placed. It was printed using the same printer previously described.

The integrating sphere (IS200-4 Thorlabs Inc., Newton, NJ, USA) and the cartridge containing the agarose film are placed in a supporting frame, designed for containing both. As an excitation source, there is a halogen lamp (HL-2000-HP Ocean Optics Inc., Orlando, FL, USA) whose light was recorded with a spectrophotometer (USB4000, Ocean Optics Inc., Orlando, FL, USA) using an integration time of 800 milliseconds, with 6 individual scans to be averaged and an adjacent average of 10 pixels (boxcar width). The spectrophotometer and the excitation source are connected to the integrating sphere through optical fibers (UV/SR-VIS High OH content, Ocean Optics Inc., Orlando, FL, USA).

The settings for the connection of the optical fiber from the light source to the sphere depends on the measurements carried out, in other words, the transmittance and reflectance, respectively. For the transmittance measurement, the light source is fixed to a slot on the underside of the supporting frame below the sample, see Figure 9a. To measure the reflectance, the light source is connected to the top port of the integrating sphere. The spectrophotometer is connected to the port perpendicularly to the light source in both configurations, as seen in Figure 9b. In Figure 9c, we show the physical experimental set-up for the optical characterization.

## Figures and Tables

**Table 1:** Descriptive statistics of the observed optical coefficients at the 809 nm wavelength of the absorption and scattering of the agarose samples with different milk concentrations.

Coefficients (1/mm)	Milk Concentration (mg/mL)			*p*-Value
	80	120	160	
$\mu_{s}^{\prime }$	0.784 ± 0.048	0.894 ± 0.060	1.021 ± 0.069	<0.0001
$\mu_{a}$	0.014 ± 0.003	0.016 ± 0.003	0.018 ± 0.004	<0.0001

## Data Availability

The data presented in this study are openly available in article.

## References

[1] Sakudo A. (2016). Near-infrared spectroscopy for medical applications: Current status and future perspectives. Clin. Chim. Acta.

[2] Pence I., Mahadevan-Jansen A. (2016). Clinical instrumentation and applications of Raman spectroscopy. Chem. Soc. Rev..

[3] Genina E.A., Bashkatov A.N., Tuchina D.K., Dyachenko P.A., Navolokin N., Shirokov A., Khorovodov A., Terskov A., Klimova M., Mamedova A. (2019). Optical properties of brain tissues at the different stages of glioma development in rats: Pilot study. Biomed. Opt. Express.

[4] Wang H., Magnain C., Sakadžić S., Fischl B., Boas D.A. (2017). Characterizing the optical properties of human brain tissue with high numerical aperture optical coherence tomography. Biomed. Opt. Express.

[5] Rajan V., Varghese B., Van Leeuwen T.G., Steenbergen W. (2009). Review of methodological developments in laser Doppler flowmetry. Lasers Med. Sci..

[6] Hoshi Y., Yamada Y. (2016). Overview of diffuse optical tomography and its clinical applications. J. Biomed. Opt..

[7] Briers D., Duncan D.D., Hirst E., Kirkpatrick S.J., Larsson M., Steenbergen W., Stromberg T., Thompson O.B. (2013). Laser speckle contrast imaging: Theoretical and practical limitations. J. Biomed. Opt..

[8] Sandell J.L., Zhu T.C. (2011). A review of in-vivo optical properties of human tissues and its impact on PDT. J. Biophotonics.

[9] Chao S.-L., Chen K.-C., Lin L.-W., Wang T.-L., Chong C.-F. (2013). Ultrasound Phantoms Made of Gelatin Covered with Hydrocolloid Skin Dressing. J. Emerg. Med..

[10] Elvira L., Durán C., Higuti R.T., Tiago M.M., Ibáñez A., Parrilla M., Valverde E., Jiménez J., Bassat Q. (2019). Development and Characterization of Medical Phantoms for Ultrasound Imaging Based on Customizable and Mouldable Polyvinyl Alcohol Cryogel–Based Materials and 3-D Printing: Application to High-Frequency Cranial Ultrasonography in Infants. Ultrasound Med. Biol..

[11] Chen S.J.-S., Hellier P., Marchal M., Gauvrit J.-Y., Carpentier R., Morandi X., Collins D.L. (2012). An anthropomorphic polyvinyl alcohol brain phantom based on Colin27 for use in multimodal imaging. Med. Phys..

[12] Nomoni M., May J.M., Kyriacou P.A. (2020). Novel Polydimethylsiloxane (PDMS) Pulsatile Vascular Tissue Phantoms for the In-Vitro Investigation of Light Tissue Interaction in Photoplethysmography. Sensors.

[13] Zell K., Sperl J.I., Vogel M.W., Niessner R., Haisch C. (2007). Acoustical properties of selected tissue phantom materials for ultrasound imaging. Phys. Med. Biol..

[14] Mustari A., Nishidate I., Wares M.A., Maeda T., Kawauchi S., Sato S., Sato M., Aizu Y. (2018). Agarose-based Tissue Mimicking Optical Phantoms for Diffuse Reflectance Spectroscopy. JoVE.

[15] Azimbagirad M., Grillo F.W., Hadadian Y., Carneiro A.A.O., Murta L.O. (2021). Biomimetic phantom with anatomical accuracy for evaluating brain volumetric measurements with magnetic resonance imaging. J. Med. Imaging.

[16] Durmuş H.O., Karaböce B., Seyidov M.Y. (2023). Experimental and Comparative Study of Optical Properties of Different Phantoms by the Kubelka–Munk Function Approach. J. Appl. Spectrosc..

[17] Thulliez M., Bastin O., Nonclercq A., Delchambre A., Reniers F. (2021). Gel models to assess distribution and diffusion of reactive species from cold atmospheric plasma: An overview for plasma medicine applications. J. Phys. D Appl. Phys..

[18] Chen Z.-J., Gillies G.T., Broaddus W.C., Prabhu S.S., Fillmore H., Mitchell R.M., Corwin F.D., Fatouros P.P. (2004). A realistic brain tissue phantom for intraparenchymal infusion studies. J. Neurosurg..

[19] Liu Y., Paliwal S., Bankiewicz K.S., Bringas J.R., Heart G., Mitragotri S., Prausnitz M.R. (2010). Rheometric Studies on Agarose Gel—A Brain Mimic Material. AAPS PharmSciTech.

[20] Tejo-Otero A., Fenollosa-Artés F., Achaerandio I., Rey-Vinolas S., Buj-Corral I., Mateos-Timoneda M.Á., Engel E. (2022). Soft-Tissue-Mimicking Using Hydrogels for the Development of Phantoms. Gels.

[21] Aernouts B., Van Beers R., Watté R., Huybrechts T., Jordens J., Vermeulen D., Van Gerven T., Lammertyn J., Saeys W. (2015). Effect of ultrasonic homogenization on the Vis/NIR bulk optical properties of milk. Colloids Surf. B Biointerfaces.

[22] Amidi E., Yang G., Uddin K.M.S., Wahidi R., Zhu Q. (2019). Low-cost ultrasound and optical gelatin-based phantoms. Proceedings of the Photons Plus Ultrasound: Imaging and Sensing 2019.

[23] Drakos T., Antoniou A., Evripidou N., Alecou T., Giannakou M., Menikou G., Constantinides G., Damianou C. (2021). Ultrasonic Attenuation of an Agar, Silicon Dioxide, and Evaporated Milk Gel Phantom. J. Med. Ultrasound.

[24] Dunn A.K. (2012). Laser Speckle Contrast Imaging of Cerebral Blood Flow. Ann. Biomed. Eng..

[25] Davoodzadeh N., Cano-Velázquez M.S., Halaney D.L., Jonak C.R., Binder D.K., Aguilar G. (2019). Optical Access to Arteriovenous Cerebral Microcirculation through a Transparent Cranial Implant. Lasers Surg. Med..

[26] Bachir W., Khir R. (2022). Characterization of pasteurized milk in the near infrared range for construction of tissue-mimicking optical phantoms. Opt. Mater. X.

[27] Pogue B.W., Patterson M.S. (2006). Review of tissue simulating phantoms for optical spectroscopy, imaging and dosimetry. J. Biomed. Opt..

[28] Liu G., Huang K., Jia Q., Liu S., Shen S., Li J., Dong E., Lemaillet P., Allen D.W., Xu R.X. (2018). Fabrication of a multilayer tissue-mimicking phantom with tunable optical properties to simulate vascular oxygenation and perfusion for optical imaging technology. Appl. Opt..

[29] Wagnières G., Cheng S., Zellweger M., Utke N., Braichotte D., Ballini J.-P., Bergh H.V.D. (1997). An optical phantom with tissue-like properties in the visible for use in PDT and fluorescence spectroscopy. Phys. Med. Biol..

[30] Augustin M.A., Clarke P.T., Craven H. (2003). POWDERED MILK | Characteristics of Milk Powders. Encyclopedia of Food Sciences and Nutrition.

[31] Huang Y., Min S., Duan J., Wu L., Li Q. (2014). Identification of additive components in powdered milk by NIR imaging methods. Food Chem..

[32] Mossoba M.M., Yurawecz M.P., Delmonte P., Kramer J.K.G. (2004). Overview of Infrared Methodologies for trans Fat Determination. J. AOAC Int..

[33] Bettati P., Chalian M., Huang J., Dormer J.D., Shahedi M., Fei B. (2020). Augmented reality-assisted biopsy of soft tissue lesions. Proceedings of the Medical Imaging 2020: Image-Guided Procedures, Robotic Interventions, and Modeling.

[34] Jacques S.L. (2013). Optical properties of biological tissues: A review. Phys. Med. Biol..

[35] Yaroslavsky A.N., Schulze P.C., Yaroslavsky I.V., Schober R., Ulrich F., Schwarzmaier H.-J. (2002). Optical properties of selected native and coagulated human brain tissues in vitro in the visible and near infrared spectral range. Phys. Med. Biol..

[36] Prahl S. Everything I Think You Should Know about Inverse Adding-Doubling. https://omlc.org/software/iad/manual.pdf.

[37] Prahl S.A., Van Gemert M.J.C., Welch A.J. (1993). Determining the optical properties of turbid media by using the adding–doubling method. Appl. Opt..

[38] Madsen S.J., Wilson B.C., Madsen S.J. (2013). Optical Properties of Brain Tissue. Optical Methods and Instrumentation in Brain Imaging and Therapy.

[39] Johansson J.D. (2010). Spectroscopic method for determination of the absorption coefficient in brain tissue. J. Biomed. Opt..

[40] Azimipour M., Baumgartner R., Liu Y., Jacques S.L., Eliceiri K., Pashaie R. (2014). Extraction of optical properties and prediction of light distribution in rat brain tissue. J. Biomed. Opt..

[41] Moffitt T., Chen Y.-C., Prahl S.A. (2006). Preparation and characterization of polyurethane optical phantoms. J. Biomed. Opt..

